# Healthcare provider-related perceptions toward deprescribing inappropriate medications among older adult outpatients

**DOI:** 10.1371/journal.pone.0312762

**Published:** 2024-11-12

**Authors:** Mohammad Jamil Rababa, Ali Al Ghazo

**Affiliations:** Department of Adult Health Nursing, Faculty of Nursing, Jordan University of Science and Technology, Irbid, Jordan; Birjand University of Medical Sciences, ISLAMIC REPUBLIC OF IRAN

## Abstract

**Objectives:**

To examine healthcare provider-related perceptions toward deprescribing inappropriate medications among older adults.

**Methods:**

A cross-sectional, correlational study used a convenience sample of outpatient older adults to measure their perception toward deprescribing using a Patient’s Perceptions of Deprescribing (PPoD), which include 57 multiple-choice questions related to patients’ sociodemographic data, health, medicines, healthcare providers, and experience of care provided by the clinic. Data were collected by a graduate nursing student from one pharmacy in a public hospital, five days per week, via in-person interviews.

**Results:**

Data were analyzed for 200 participants. The level of patient collaboration with their primary care providers (PCPs) is linked to their trust in PCPs, beliefs about medication use, PCP knowledge, and medication concerns (p < .0001). Patient involvement in medication deprescribing decision-making is also associated with trust in PCPs and willingness to stop a medication (p < .0001). Additionally, trust in PCPs is related to patient involvement in decision-making, PCP knowledge, general health, collaboration with PCPs, and receiving conflicting information about a medicine (p = .010). Lastly, PCP medication knowledge is associated with trust in PCPs, views on the importance of medicines, medication concerns, seeking help with medicines, interactions with clinical pharmacists, and being advised by a clinical pharmacist to discontinue medication (p < .0001).

**Conclusions:**

The study found that older adults’ trust in their PCP, collaboration with their PCP, involvement in the decision-making of deprescribing, and knowledge about medication are associated with clinical and medicine-related factors. Therefore, PCPs should discuss the benefits of deprescribing inappropriate medications to prevent long-term side effects. Future studies should focus on the effectiveness of evidence-based deprescribing protocols for older adults.

## Introduction

Inappropriate medication prescribing for older adults is a common problem [1, 2]. A study conducted recently found that the prevalence of inappropriate prescribing of medications for older adults is as high as 40% in community health centers [1]. This type of prescribing can lead to drug interactions, drug-nutrient interactions, and iatrogenic diseases, which can all contribute to an increase in the rates of morbidity and mortality as well as increased healthcare costs [3, 4]. For example, in Ireland, the expenditure on potentially inappropriate prescribing was projected to be €45,631,319 in 2007 [5]. Consequently, the appropriate management for potentially inappropriate prescribing contributes to a cost reduction of US$ 193 to US$ 4,966 per patient annually for experimental studies and US$ 3 to US$ 2,505 per patient annually for observational studies across 3,662 articles systematically reviewed [1].

A recent systematic review reported that potentially inappropriate medications were estimated to affect 371.2 million older adults from 17 countries [6]. Based on a prospective analysis conducted by Khezrian et al. revealed that around one-third of older adults (37%) took at least five prescription medications per day, and around half of older adults (47%) took one or more inappropriate or unnecessary medications [7]. Developing countries have been noted to have higher potentially inappropriate medication prevalence compared to developed countries [6]. In the previous Jordanian study, the using unnecessary medications constituted a third of the total number of medications used by elderly patients [8].

Deprescribing is the process of stopping or reducing the dosage of an improper/inappropriate drug under a doctor’s supervision [9]. Age-related pharmacodynamic and pharmacokinetic changes may increase medication sensitivity in older adults. As such, deprescribing can be essential to care for the older adult [10]. It is also considered a practical way to support evidence-based and person-centered care [11]. Unfortunately, data suggests that one in five medications taken by an older adult is inappropriate where medication risks may exceed the benefits or medications do not align with the patient’s preferences and treatment goals. This suggests that deprescribing might not occur as frequently as it should in practice [12]. According to the WHO guidelines for medication prescribing, patients should actively participate in the prescribing process [13, 14]. Consequently, patients should also be central to medication deprescribing [13, 14]. Patient outcomes may improve due to initiatives to enhance patient-centered care in practice [13, 14]. Patient participation and involvement are central tenets of patient-centered care, including honoring and adjusting treatment to patients’ needs, preferences, and values [15]. Therefore, patients’ beliefs, attitudes, perceptions, and preferences must be ascertained before putting this into action.

Two-thirds of older adults prefer to discontinue one medication if it is medically possible and desire to do so more than their healthcare providers [16]. Despite satisfaction with current medications being considered a pivotal factor in deprescribing drugs [16], numerous factors should be investigated among older adults when deprescribing drugs. Healthcare providers believe many inappropriate medications should be reviewed and deprescribed [17]. Although this is not in accord with what older adults and their care providers thought, these medications are necessary, and there is no need to reduce their number [16, 17]. However, older adults strongly believe it is crucial to involve them in the decision-making regarding the number of medications and whether the prescribing drugs should be changed [18].

It has been shown that older adults’ willingness to reduce the number of medications and the change in deprescribing drugs is based on numerous variables, such as perception of the inappropriateness of drugs prescribed to these people for stopping medications [18]. Moreover, many factors are getting in the way of deprescribing medications among older adults, such as lack of knowledge, time, miscommunication, abandonment of care perception, concerns about adverse effects, and older adults’ resistance [19]. However, no previous study has explored healthcare provider-related perceptions toward deprescribing inappropriate medications among older adults. This study aimed to investigate these perceptions and their associated factors among older adults.

## Materials and methods

### Design

A cross-sectional, correlational design was used in this study to examine healthcare provider-related perceptions toward deprescribing inappropriate medications among older adults.

### Sampling technique and sample size calculation

In this study, a convenience sampling method was used to recruit patients aged 55 and older from an outpatient clinic in Irbid, Jordan. The older adult age is defined at 55 years old in Jordan [20]. The sample size of 200 participants was determined using a G-power analysis, considering an alpha level of 0.05, an anticipated medium effect size of 0.15, a desired statistical power of 90%, and 13 predictors.

### Inclusion and exclusion criteria

Older adults taking at least one PIM according to the updated Beers criteria were eligible for inclusion in this study. Additionally, they had to be able to provide informed consent and communicate in Arabic. The following people were excluded from the study: (1) Patients who are unable to provide reliable information or comprehend the study due to severe cognitive impairment or dementia, (2) patients with significant hearing or visual impairment that would hinder communication with the study team, (3) and patients with serious illnesses in a terminal stage.

### Data collection

Ethical Approval was obtained from the Institutional Review Board (IRB) of Jordan University of Science and Technology (IRB #378–2023). Each participant was informed about the purpose of the study, its significance, the estimated time required to complete the interview, and their right to withdraw without any penalties or explanation. Written Informed consent was obtained from each participant, and an anonymous questionnaire was used to maintain privacy and confidentiality. Data were collected by a graduate nursing student from a single pharmacy in a public hospital, conducting in-person interviews five days a week, from August 1st to December 30th, 2023. Data collection was supervised by a nursing associate professor, and extensive training was provided to the data collector on communicating with older adults, administrating the questionnaire, and calculating the scores. The reliability and accuracy of the collected data were checked by the researcher every 10% of the questionnaire. A demographic data sheet was used to collect participants’ sociodemographic and clinical data.

The Patient’s Perceptions of Deprescribing (PPoD), which Linsky et al. developed [21], was used to measure the participant’s perception of deprescribing inappropriate medications. The questionnaire includes 57 multiple-choice items related to patients’ sociodemographic data, experience of care provided by the clinic, medicines, health, and healthcare providers. Patients were asked to rate their perceptions related to patients’ medicines, health, and healthcare provider items on a 5-point Likert scale ranging from 1 = "strongly disagree" to 5 = "strongly agree." The responses were then scored and analyzed to evaluate patients’ perceptions of medication deprescribing and their willingness to be involved in the deprescribing process. The questionnaire consists of eight subscales, including medication concerns (6 items), provider knowledge (3 items), interest in stopping medicines (3 items), patient involvement in DM (3 items), unimportance of medications (3 items), belief about medication (4 items), trust in healthcare providers (5 items), and collaboration with healthcare providers (3 items). The subscale scores were computed as the items mean. For this study, only the scores of subscales related to healthcare providers (knowledge, trust, collaboration, and involvement in DM) were analyzed. The PPoD is a valid and reliable tool. The Cronbach α of the questionnaire subscales in the current study article were as follows: trust (0.94), provider knowledge (0.94), involvement (0.85), and collaboration (0.94).

The PPoD was translated into Arabic by two associate professors of English and nursing, and back-translated into English by a linguistics professor. No significant differences were found between the two versions. The two English versions were thoroughly reviewed to reach an agreement on minor discrepancies.

An associate professor in geriatrics and two professors in clinical pharmacology were consulted to review the translated version of the questionnaire for its face and content validity in terms of its equivalent meaning, cultural relevance, and clarity. The survey’s content validity index was 0.95, with no recommendations to remove or add any item. All the professors agreed that all the items of the translated questionnaire were relevant and equivalent in meaning to the original version. The items are clear and culturally relevant.

A pilot study was carried out from July 24th to July 29th, 2023, to assess the feasibility of the study and to evaluate the feasibility, clarity, and reliability of the Arabic version of the questionnaire. This pilot study involved 12 older adults, 12% of the total sample. The researchers conducted 15-minute face-to-face interviews with the participants in a quiet, private room to gather the necessary data. During the interviews, the participants were asked to express their thoughts about the clarity of the questionnaire items. The participants reported no difficulty in understanding and responding to the questionnaire items. The data collected in the pilot study was not included in the analysis of the original study data. Moreover, data collection for the original study was also done through face-to-face interviews in a private, quiet room by a well-trained data collector with a master’s degree in nursing. The average interview duration was 15 minutes.

### Data analysis

The continuous sociodemographic variables were described using the mean and standard deviation, while the categorical ones were described using frequency and percentages. The description of variables measured with more than one option, like comorbidity was analyzed by the Multiple-response dichotomy analysis. The statistical normality of metric-measured variables was tested by the Histogram and Kolmogrove-Smirnove. Cronbach’s alpha was used to evaluate the reliability of the PPoD questionnaire subscales through an internal consistency test. The multivariable linear regression analysis was utilized to explore the significant predictors of the four PPoD subscale scores, including trust in PCP and PCP knowledge, collaboration with PCP, and involvement of patients in DM. The relationship between independent predictor variables and dependent outcome variables was analyzed using unstandardized beta coefficients and 95% confidence intervals. Outliers were identified and excluded from regression analysis using residual analysis, Mahalanobis distance, and beta indices. Data analysis was conducted using IBM SPSS Statistics software version 28, with a significance level of 0.050. The dataset is outlined in S1 Dataset.

## Results

### Descriptive analysis of patient’s socio-demographic characteristics

Two hundred older adults were enrolled in the study and completed and returned the study questionnaire. Table 1 displays the patients’ socio-demographic and clinical characteristics. Most participating older adults were males (55%), married (65%), and completing primary school education. Most of the older adults had several medical and psychiatric comorbid problems, such as anxiety disorder (9.3%), diabetes mellitus (19.5%), and hypertension (21.7%).

**Table 1:** Descriptive analysis of patients’ socio-demographic characteristics. N = 200.

	Frequency	Percentage
Sex		
Male	110	55
Female	90	45
Age group		
46–55 years	27	13.5
56–65 years	46	23
66–75 years	64	32
76–85 years or more	63	31.5
Marital state		
Single, never married	7	3.5
Married	130	65
Divorced	10	5
Widowed	53	26.5
Highest Level of education		
Primary school	92	46
Completed high school	27	13.5
Vocational or technical training	13	6.5
Community college	33	16.5
Bachelor’s degree	32	16
Graduate studies	3	1.5
Comorbidity, n = 199.		
Anxiety	72	9.3
Arthritis	118	5.3
Cancer	23	3
Chronic Lung Disease	57	7.4
Depression	16	2.1
Diabetes	150	19.5
Heart Failure	58	7.5
Hypertension	167	21.7
Ischemic Heart Disease	83	10.8
Post-traumatic Stress Disorder	21	2.7
Stroke	6	0.8

### Predictors of patients’ perceived collaboration with PCP

The multivariable linear regression analysis was applied to examine patients’ perceived collaboration with PCP on medication deprescribing and its associated variables. As shown in Table 2, a statistically significant regression equation was found (p < .0001). Almost half of the variables entered into the regression model were statistically significant predictors (p < .05). For example, the analysis model showed that patients’ perceived trust in their PCP provider is associated significantly and positively with their mean perceived collaboration with PCP, beta coefficient = 0.428, p value = 0.001, indicating a higher level of trust in PCP predicts significantly higher perceived collaboration with PCP.

**Table 2:** Multivariable linear regression analysis of patients’ perceived collaboration with care provider.

	Unstandardized Beta Coefficients	95.0% C. I for Beta coefficient		
		Lower Bound	Upper Bound	p-value
(Constant)	0.685	-1.190	2.560	0.472
Sex: female vs male	-0.001	-0.235	0.232	0.990
Age group	0.000	-0.113	0.113	0.999
Patient involvement in DM	-0.086	-0.232	0.059	0.242
Interest in stopping medicines	-0.105	-0.319	0.110	0.336
Trust in PCP	0.428	0.182	0.674	0.001
Beliefs about medication overuse	0.223	0.044	0.402	0.015
PCP knowledge	0.227	0.018	0.436	0.033
Medications concern	0.237	0.079	0.395	0.004
Unimportance of medicines	-0.187	-0.441	0.068	0.150
Have you ever asked your PCP if you could stop taking a medicine?	-0.583	-0.866	-0.301	<0.001
perceived General Health (GH)	0.072	-0.052	0.195	0.253

Dependent outcome variable: Patients’ perceived collaboration with providers. Model R-squared = 0.294, adjusted R-squared = 0.252. Model overall significance: f (11,188) = 7.1, p-value<0.001.

### Predictors of patients’ perceived trust in PCP

Table 3 displays the multivariable linear regression analysis of patients’ perceived trust in their PCP provider and associated factors. As shown in Table 3, a statistically significant regression equation was found (p < .0001). Almost half of the variables entered into the regression model were statistically significant predictors (p < .05). For example, patients’ perception of their PCP’s medication knowledge is associated positively and significantly with their perceived trust in PCP, beta coefficient = 0.172, p value = 0.004. This indicates that higher levels of perceived PCP medication knowledge are associated with higher levels of patients’ perceived trust in PCP.

**Table 3:** Multivariable linear regression analysis of patients’ perceived trust in PCP.

	Unstandardized Beta Coefficients	95.0% C. I for Beta coefficient		
		Lower Bound	Upper Bound	p-value
(Constant)	3.831	2.884	4.778	<0.001
Sex: female vs male	-0.089	-0.214	0.036	0.162
Age group	-0.019	-0.081	0.043	0.546
Perceived Beliefs about Medication Overuse	0.082	-0.023	0.187	0.125
Perceived Interest in Stopping Medicines	-0.019	-0.137	0.098	0.747
Perceived Medications concerns	-0.009	-0.096	0.078	0.837
Perceived Unimportance of Medicines	-0.070	-0.208	0.067	0.315
Perceived Patient involvement in DM	-0.095	-0.174	-0.015	0.020
Perceived PCP knowledge	0.172	0.055	0.289	0.004
Self-rated general health (GH)	-0.074	-0.142	-0.007	0.032
Perceived Collaboration with PCP.	0.137	0.065	0.209	<0.001
Have you ever had a PCP tell you one thing about a medicine and another one tell you something different?	-0.168	-0.296	-0.040	0.011
Imagine that your PCP prescribed a medicine for you. Would you be comfortable if a clinical pharmacist told you to stop taking it?	0.153	-0.011	0.316	0.068
Have you seen the clinical pharmacist provider in the past 12 months?	-0.243	-0.406	-0.080	0.004

Dependent outcome variable: Patients’ overall perceived trust in care provider score. Model R-squared = 0.365, adjusted R-squared = 0.321. Model significance: f (13,186) = 8.24, p-value<0.001.

### Predictors of patients’ perceived involvement in decision-making

The multivariable regression analysis of the patients’ mean perceived involvement in DM score showed a statistically significant regression equation (p < .0001). As seen in Table 4, only two variables entered into the regression model were statistically significant predictors (p < .05). For example, patients’ perceived trust in their PCP is associated negatively and significantly with their perception regarding involvement in medications DM, beta coefficient = -0.244, p-value = 0.050. As patients’ trust in their PCP increased, their mean perceived involvement in their medication DM score decreased accordingly.

**Table 4:** Multivariable linear regression analysis of patients’ perceived patient involvement in DM.

	Unstandardized Beta Coefficients	95.0% C. I for Beta coefficient		
		Lower Bound	Upper Bound	p-value
(Constant)	2.815	1.007	4.623	0.002
gender: female vs male	0.017	-0.206	0.240	0.879
Patients age group	-0.029	-0.139	0.082	0.609
Beliefs about Medication overuse	0.003	-0.174	0.180	0.975
Interest in stopping medicines	-0.183	-0.390	0.023	0.082
PCP knowledge	0.150	-0.058	0.358	0.156
Medications concern	0.122	-0.028	0.272	0.111
Unimportance of medicines	0.145	-0.106	0.396	0.256
Trust in PCP	-0.244	-0.488	0.000	0.050
Imagine that your PCP prescribed a medicine for you. Would you be comfortable if a clinical pharmacist told you to stop taking it?	0.407	0.117	0.697	0.006
Collaboration with PCP	-0.100	-0.233	0.033	0.138
Self-rated general health	0.039	-0.081	0.159	0.521
How many prescription medicines have you filled at a pharmacy outside the clinic?	0.118	-0.010	0.246	0.071

The dependent outcome variable is the patients’ overall mean perceived patient involvement in decision-making score. The Model R-squared is 0.128, and the adjusted R-squared is 0.072. The model significance is f (12,187) = 2.283, and the p-value is 0.010.

### Predictors of patients’ perceived PCP medication knowledge

The multivariable linear regression analysis of patients’ perceived PCP knowledge about medicine is shown in Table 5. The analysis yielded a statistically significant regression equation (p = .010). The majority of the variables entered into the regression model were statistically significant predictors (p < .05). For example, patients’ perceived collaboration with PCP is associated significantly and positively with their perceived PCP knowledge about medicine, indicating greater collaboration with PCP predicts significantly higher patients’ perceived PCP knowledge about medicine, beta coefficient = 0.095, p-value = 0.034.

**Table 5:** Multivariable linear regression analysis of patients’ perceived PCP medication knowledge.

	Unstandardized Beta Coefficients	95.0% C. I for Beta coefficient		
		Lower Bound	Upper Bound	p-value
(Constant)	2.593	1.529	3.657	<0.001
Gender	0.013	-0.135	0.161	0.862
Age	-0.057	-0.128	0.014	0.114
Collaboration with PCP	0.095	0.007	0.182	0.034
Trust in PCP	0.262	0.099	0.425	0.002
Unimportance of medicines	-0.008	-0.172	0.156	0.921
Patient involvement in DM	0.073	-0.022	0.167	0.133
Interest in stopping medicines	0.008	-0.130	0.147	0.908
Medications concerns	-0.109	-0.205	-0.014	0.025
Beliefs about Medication Overuse	-0.026	-0.151	0.098	0.679
Receiving help taking medicines from friends/family members (any help).	0.269	0.059	0.479	0.012
Have you seen the clinical pharmacists in the past 12 months?	0.246	0.055	0.438	0.012
Imagine that your PCP prescribed a medicine for you. Would you be comfortable if a clinical pharmacist told you to stop taking it?	0.319	0.129	0.510	0.001

Dependent outcome variable: Patients’ overall mean perceived PCP knowledge in medications score. Model R-squared = 0.246, adjusted R-squared = 0.195. Model significance: f (12,187) = 5.10, p-value<0.001.

## Discussion

The current study’s findings highlighted older adults’ perceptions of medication deprescribing related to PCP knowledge, trust in PCP, collaboration with PCP, and involvement in DM. This is the first study to examine these perceptions among older adults in Jordan and the region.

### Factors associated with patients’ perceived collaboration with PCP

Our study findings revealed that patients’ trust in PCP, beliefs about medication overuse, PCP knowledge, medication concerns, and asking doctors to stop their medications had a statistical impact on older adults’ perceived collaboration with PCP. These findings highlight the multi-faceted nature of the relationship between PCP and older adults. Addressing medication-related beliefs and concerns, building trust, facilitating patient involvement in DM, and ensuring provider knowledge are crucial aspects that can enhance the perceived collaborative care experienced by older adults [18, 22]. Therefore, communication strategies and healthcare interventions can be tailored to identify these specific factors and improve overall patient satisfaction and engagement [18, 22]. Due to the limited studies that investigated our topic using the PPoD questionnaire, our findings in this section were justified based on previous studies on the role of collaboration with PCP in improving patients’ health and care quality regarding medication deprescribing, such as those conducted by Linsky et al. [23], Gerlach et al. [24], Buzancic et al. [25], Lundby et al. [26], and Trenaman et al. [27].

### Factors associated with patients’ perceived trust in PCP

Our findings reported that patients’ involvement in DM, perceived PCP knowledge, perceived collaboration with PCP, and seeing clinical pharmacists in the last 12 months were significantly associated with patients’ trust in PCP. These findings indicate the importance of PCP knowledge, patient involvement in DM, collaborative interactions, and routine clinic/hospital visits in shaping patients’ trust. Promoting these aspects of the patient-PCP relationship can have positive implications for overall trust levels, the quality of healthcare interactions, and patient satisfaction [28–31]. These previous studies generally highlighted the critical role of patients’ trust in their PCP in their health outcomes and DM regarding their health plans. Thus, healthcare systems and professionals may adopt strategies to enhance patient communication, engagement, and collaborative DM to strengthen trust and improve patient experience.

### Factors associated with patients’ perceived involvement in decision-making

Our finding revealed that patients’ trust in PCP is significantly associated with patients’ involvement in DM. Based on these findings, it is essential to note that any successful healthcare PCP-patient relationship is built on trust [29, 31]. Patients are more likely to feel at ease giving pertinent information, discussing health issues, and following treatment plans when they have faith in their PCPs [28, 32]. As a result, trust is formed by factors including the provider’s skill, empathy, communication abilities, and apparent dedication to the patient’s well-being [28, 32]. Furthermore, trust promotes honest and efficient communication between healthcare professionals and patients [32]. Patients are more willing to ask questions, share their preferences, and have meaningful conversations about their health when they have confidence in their healthcare providers [28]. Thus, our findings indicate that patients are more likely to actively engage in the treatment plan DM process when they have confidence in their healthcare professionals. This involvement can shape DM regarding their care or even discuss available treatment alternatives [33]. Patients and clinicians work together to make decisions in shared DM that take into account the patient’s preferences, values, and particular circumstances [21, 23, 30, 34, 35].

### Factors associated with patients’ perceived care provider medication knowledge

Our findings revealed that collaboration with PCP, patients’ trust in PCP, medication concerns perceptions, receiving help when taking medications, and seeing clinical pharmacists in the last 12 months are significantly associated with patients’ perceived PCP medication knowledge. These findings suggest that providing comprehensive and coordinated care requires cooperation across healthcare professionals. Thus, patients’ opinions of the level of drug knowledge possessed by their PCPs can be positively impacted when PCPs collaborate and communicate well [18, 22, 24–27]. Moreover, these results imply that trust is critical to the relationship between patients and PCPs. Patients are more inclined to believe in the competency of PCP—including their knowledge of medications—when they have faith in them. As a result, establishing and preserving trust requires effective communication, empathy, and knowledge acquisition. Patients’ trust in the medication knowledge of their PCPs might be positively impacted by trust [10, 29, 31].

Regarding patients’ perceptions about medication concerns, these findings suggest that patients’ perceptions of PCP medication knowledge can be improved by attending to and acknowledging their concerns, giving them clear information, and participating in DM. Open communication regarding the possible risks, advantages, and side effects of drugs might, therefore, help to foster a more positive and educated perspective [18, 22, 26, 27]. Finally, these findings also indicate that older adults who receive support and assistance when taking their medications may have a more positive perception of their PCP knowledge. This assistance can include addressing concerns, guidance on proper administration, and providing educational resources. On the other hand, these findings indicate that clinical pharmacists’ involvement in older adult care can be associated significantly with medication management. Thus, with their specialized medication knowledge, healthcare providers can identify concerns, collaborate with other healthcare providers, and provide education.

### The implications of the study findings

This study has several implications for policymakers, healthcare administrators, nursing education, and clinical practices.

#### Implications for policymakers and healthcare administrators

The findings of the current study could contribute to fostering a trust-based relationship between healthcare providers and their patients. This relationship is a crucial factor in successfully applying the medication deprescribing intervention [36]. It simplifies the task for healthcare providers and boosts patients’ acceptance of the intervention, thereby increasing their adherence to the guidelines and recommendations of medication prescription.

The study findings advocate for a significant shift in the healthcare paradigm, one that places increased patient involvement in the decision-making process at its core. This shift is crucial in promoting patient-centered care and enhancing patient involvement in their health and deprescribing intervention. The current study findings imply that policymakers, healthcare administrators, and healthcare providers should focus on enhancing patients’ knowledge and awareness about their medications and health. This can be achieved through training workshops and educational sessions highlighting the significance of deprescribing intervention on patients’ health outcomes and economics.

#### Implications for clinical practice

The findings of the current study could contribute to holding training workshops to increase nurses’ awareness and knowledge regarding medication deprescribing and its importance to patients’ health and safety and the quality of medical services. Conducting such workshops on an ongoing basis will enhance nurses’ clinical competencies in spreading awareness and knowledge among patients and encouraging them to begin medication deprescribing correctly to ensure their safety and health.

### Limitations

The present study included several limitations. It utilized a cross-sectional design nonprobability sampling method, and a self-reported tool may be associated with selection and reporting bias. An experimental study examining the effectiveness of an evidence-based deprescribing protocol in minimizing the clinical side effects associated with inappropriate medication prescriptions among older adults is recommended. Also, replicating this cross-sectional study with a larger sample and different inpatient categories is recommended to expand the generalizability of the findings.

## Conclusions

Inappropriate prescription of medication is one of the most common polypharmacy-related issues among older adults. The current study reported its findings on healthcare-related perceptions toward deprescribing inappropriate medications among older adults. Moreover, the study found that older adults’ perceived trust in PCP, collaboration with PCP and knowledge about medication, and involvement in DM of medication deprescribing are significantly associated with clinical (e.g., self-rated general health) and medicine-related factors (e.g., the unimportance of medications). Therefore, PCPs should discuss the benefits of deprescribing inappropriate medications with their patients to prevent the side effects associated with long-term unnecessary use. The long-term use of inappropriate medications by older adults should be carefully evaluated. Future studies on the effectiveness of an evidence-based deprescribing protocol on minimizing the clinical side effects associated with inappropriate prescription of medications among older adults are recommended.

## Supporting information

S1 Dataset PRISMA 2020 checklist. (XLSX)
